# Parathyroid hormone-related protein secretion is inhibited by oestradiol and stimulated by antioestrogens in KPL-3C human breast cancer cells.

**DOI:** 10.1038/bjc.1997.310

**Published:** 1997

**Authors:** J. Kurebayashi, H. Sonoo

**Affiliations:** Department of Breast and Thyroid Surgery, Kawasaki Medical School, Okayama, Japan.

## Abstract

We recently established a human breast cancer cell line, KPL-3C, from a breast cancer patient with humoral hypercalcaemia. This cell line possesses oestrogen receptor (ER) and secretes parathyroid hormone-related protein (PTHrP) into medium. To investigate the effects of oestrogen and antioestrogens on PTHrP secretion, KPL-3C cells were cultured for 48 h in an oestrogen-eliminated medium with 17beta-oestradiol (E2), tamoxifen (TAM) and/or a pure antioestrogen, ICI182,780 (ICI), and PTHrP secretion was measured using an immunoradiometric assay. The effects of these agents on cell cycle progression were also studied using flow cytometry. E2 (1-100 nM) significantly inhibited PTHrP secretion, whereas both TAM (0.1-10 microM) and ICI (1-100 nM) significantly stimulated it. These effects were completely blocked by the simultaneous addition of 1 nM E2 to the medium. At the same time, E2 significantly increased the percentage of cells during the S and G2/M phases, whereas both antioestrogens significantly increased the percentage of cells during the G0/G1 phase. Again, these cytostatic effects were completely reversed by the addition of E2. These findings indicate that antioestrogens inhibit the growth of ER-positive breast cancer cells but may stimulate PTHrP secretion and that these effects may be mediated by ER.


British Journal of Cancer (1997) 75(12), 1819–1825
© 1997 Cancer Research Campaign

### Parathyroid hormone-related protein secretion is inhibited by oestradiol and stimulated by antioestrogens in KPL-3C human breast cancer cells

**J Kurebayashi and H Sonoo**

Department of Breast and Thyroid Surgery, Kawasaki Medical School, 577 Matsushima, Kurashiki, Okayama 701-01, Japan

**Summary** We recently established a human breast cancer cell line, KPL-3C, from a breast cancer patient with humoral hypercalcaemia. This cell line possesses oestrogen receptor (ER) and secretes parathyroid hormone-related protein (PTHrP) into medium. To investigate the effects of oestrogen and antioestrogens on PTHrP secretion, KPL-3C cells were cultured for 48 h in an oestrogen-eliminated medium with 17β-oestradiol ($E_2$), tamoxifen (TAM) and/or a pure antioestrogen, ICI182,780 (ICI), and PTHrP secretion was measured using an immunoradiometric assay. The effects of these agents on cell cycle progression were also studied using flow cytometry. $E_2$ (1–100 nM) significantly inhibited PTHrP secretion, whereas both TAM (0.1–10 μM) and ICI (1–100 nM) significantly stimulated it. These effects were completely blocked by the simultaneous addition of 1 nM $E_2$ to the medium. At the same time, $E_2$ significantly increased the percentage of cells during the S and $G_2$/M phases, whereas both antioestrogens significantly increased the percentage of cells during the $G_0$/$G_1$ phase. Again, these cytostatic effects were completely reversed by the addition of $E_2$. These findings indicate that antioestrogens inhibit the growth of ER-positive breast cancer cells but may stimulate PTHrP secretion and that these effects may be mediated by ER.

**Keywords:** parathyroid hormone-related protein; breast cancer; antioestrogen; oestrogen receptor; cell cycle

Parathyroid hormone-related protein (PTHrP) was first purified from culture medium obtained from a human lung cancer cell line, established from a hypercalcaemic patient, and its complementary DNA was cloned by the same group (Moseley et al, 1987; Suva et al, 1987). PTHrP secreted by malignant cells has been proved to be the main cause of humoral hypercalcaemia associated with malignancy (Burtis et al, 1990; Grill et al, 1991; Ratcliffe et al, 1992). In this situation, PTHrP acts on kidney and bone as a circulating hormone like parathyroid hormone and subsequently induces hypercalcaemia. The PTHrP secreted by cancer cells act as autocrine and paracrine mediators that stimulate the growth of cancer cells (Kaiser et al, 1992; Benitez-Verguizas et al, 1994; Iwamura et al, 1994) and activate osteoclasts to produce osteolytic metastasis (Southby et al, 1990; Bundred et al, 1991; Powell et al, 1991; Birch et al, 1995). In addition, recent reports suggest that PTHrP may play a certain role in cancer invasion and metastasis (Vargas et al, 1992; Li and Drucker, 1994; Luparello et al, 1995; Akino et al, 1996). These findings support the hypothesis that PTHrP plays a key role in malignant progression and that suppression of PTHrP production and secretion from malignancy could be an attractive strategy for cancer therapy.

Regulation of PTHrP secretion has been widely studied in cancer cell lines (Deftos et al, 1989; Emly et al, 1994; Endo et al, 1994; Merryman et al, 1994; Rizzoli et al, 1994), human T-cell lymphotrophic virus, type I-infected T cells (Inoue et al, 1993), normal and transformed keratinocytes (Henderson et al, 1991; Kremer et al, 1991; Allinson and Drucker, 1992), primary cultures of mammary epithelial cells (Thiede, 1989; Ferrari et al, 1992; Sebag et al, 1994), pituitary cells (Holt et al, 1994) and cultured amniotic fluid cells (Dvir et al, 1995). Steroid hormones, such as glucocorticoids, vitamin D and its analogues and progestins are reported to inhibit PTHrP secretion. On the other hand, it has been reported that growth factors, such as epidermal growth factor, insulin-like growth factors I and II, transforming growth factor beta (TGF-β) and peptide hormones, such as human calcitonin, prolactin and placental lactogen, stimulate PTHrP secretion. However, there are only four reports describing the effect of oestrogen on PTHrP expression or secretion. Two of these suggest that exogenous administration of 17β-oestradiol ($E_2$) stimulated PTHrP expression in rat uterus in vivo (Thiede et al, 1991; Paspaliaris et al, 1992). One of these reports suggests that $E_2$ induced a rapid and transient increase in PTHrP mRNA expression in rat pituitary cells (Holt et al, 1994); the other suggests that $E_2$ has no effect on PTHrP secretion from cultured amniotic fluid cells (Dvir et al, 1995). To the best of our knowledge, no report has described the effects of oestrogen and antioestrogens on PTHrP secretion from oestrogen receptor (ER)-positive breast cancer cells. Because immmunohistochemical, in situ hybridization or polymerase chain reaction analyses have demonstrated that 60% of primary breast cancers express PTHrP (Southby et al, 1990; Bundred et al, 1991; Powell et al, 1991; Birch et al, 1995) and ER expression is one of the most characteristic features of breast cancer, it is conceivable that there might be an oestrogenic regulation of PTHrP production and secretion in breast cancer. Therefore, the regulation of oestrogen and antioestrogens on PTHrP secretion was investigated in this study using a human breast cancer cell line, KPL-3C, which possesses both ER and progesterone receptor (PgR) and stably secretes immunoreactive PTHrP into culture medium (Kurebayashi et al, 1996). In addition, to investigate the relationship between PTHrP secretion and cell

*Received 17 May 1996*
*Revised 3 December 1996*
*Accepted 18 December 1996*

*Correspondence to:* J Kurebayashi cycle, the effect of oestrogen and antioestrogens on cell cycle progression was also analysed in this cell line.

### MATERIALS AND METHODS

### Chemicals

$E_2$ was purchased from Sigma Chemical (St Louis, MO, USA). Tamoxifen citrate (TAM) and a pure steroidal antioestrogen, ICI182,780 (ICI) were kindly provided by Zeneca Pharmaceuticals (Macclesfield, UK). A stock solution of these chemicals was prepared in 100% ethanol, with the final concentration of ethanol being 0.1%. Control cells were treated with the culture medium supplemented with 0.1% ethanol.

### Cell culture

The KPL-3C human breast cancer cell line was recently established from the malignant pleural effusion of a breast cancer patient with humoral hypercalcaemia of malignancy. The histological type of the primary tumour of this cell line was an invasive ductal carcinoma. KPL-3C cells were maintained in RPMI-1640 medium supplemented with 5% fetal calf serum. The population doubling time was approximately 72 h at the exponential growth phase (Kurebayashi et al, 1996).

For the following experiments, KPL-3C cells were plated at a density of $2 \times 10^5$ cells per well in 12-well plates in phenol red-free RPMI-1640 medium supplemented with 2% dextran-coated charcoal-stripped fetal calf serum (Scholl et al, 1983) ($E_2$-eliminated medium). When the cells became semiconfluent, the $E_2$-eliminated medium supplemented with various concentrations of $E_2$, TAM and/or ICI was added to the wells after washing twice with phosphate-buffered saline (PBS). After 48 h, conditioned medium was collected and centrifuged at 1500 *g* for 10 min to remove the contamination of floating cells, and the PTHrP concentration of the supernatant was measured by an immunoradiometric assay (IRMA) or a C-terminal-region-specific radioimmunoassay (RIA).

**Figure 1** Effects of $E_2$ and antioestrogens on PTHrP secretion from KPL-3C cells. After washing with PBS, semiconfluent KPL-3C cells were incubated with $E_2$-eliminated medium supplemented with various concentrations of agents for 48 h. Conditioned media were collected, and the concentration of PTHrP was measured by an IRMA. PTHrP secretion was calculated as described in Materials and methods. Values represent the mean percentages of control. Bars show s.d. **P* < 0.05 in comparison with control; ***P* < 0.01 in comparison with control

The treated cells were trypsinized and harvested to measure the number of cells by trypan blue exclusion and to analyse the cell cycle by flow cytometry.

### Measurement of PTHrP

The PTHrP concentration in the conditioned media was measured with a two-site IRMA kit (Mitsubishi Petrochemical, Tokyo, Japan) as described elsewhere (Ikeda et al, 1994). Briefly, a rabbit anti-human PTHrP (50–83) polyclonal antibody and a mouse anti-human PTHrP (1–34) monoclonal antibody were used, with recombinant human PTHrP (1–87) being used as the standard in this assay. The detection limit was 0.5 pM, and the coefficients of intra- and interassay variations were not higher than 7.5%. On the basis of our previous report (Kurebayashi et al, 1996), the PTHrP secretion into the medium was defined as follows:

$$\text{Secretion per cell per 48 h} = \frac{\text{concentration of PTHrP} \times \text{volume of medium}}{\text{mean cell number}}$$

The basal PTHrP secretion from the control cells was approximately 8 fmol per $/10^6$ cells per 48 h. PTHrP secretion from the treated cells was expressed as percentages of the control.

To investigate the effect of $E_2$ and antioestrogens on proteolytic processing of mature PTHrP, C-terminal PTHrP was also measured by a C-terminal-region-specific RIA kit (Daiichi Radioisotope Laboratories, Chiba, Japan) as described elsewhere (Kasahara et al, 1992). Briefly, a sheep antiserum immunized with a synthetic human PTHrP(109–141) was used, with an $^{125}$I-labelled synthetic peptide, [Tyr$^{108}$] PTHrP(108–141), being used as a tracer in this assay. Neither human PTHrP(1–34) nor human PTHrP(67–86) has been reported to cross-react in this assay. The detection limit was 2.0 pM for recombinant human PTHrP-(109–141), and the coefficients of intra- and interassay variations were 5.9% and 3.9% respectively.

### Cell cycle analysis

To investigate the effects of the agents on cell cycle progression, KPL-3C cells were trypsinized, harvested and stained with propidium iodine using the CycleTest Plus DNA Reagent Kit (Becton Dickinson, San Jose, CA, USA). Flow cytometry was performed with a FACSort flow cytometer (Becton Dickinson), and the DNA histogram was analysed by a CellFit Cell-Cycle system (Becton Dickinson).

### Statistical analysis

PTHrP secretion and the percentage of cells in the control group and treatment group during each cell cycle phase were compared using the one-way analysis of variance. All of the control and treatment groups were tested in triplicate, and each experiment was performed at least twice to confirm its reproducibility.

### RESULTS

### Regulation of PTHrP secretion by $E_2$ and antioestrogens

As shown in Figure 1, 1–100 nM $E_2$ significantly inhibited PTHrP secretion from KPL-3C cells (mean percentage of

© Cancer Research Campaign 1997

**Table 1** Effects of oestradiol and antioestrogens on cell proliferation and PTHrP secretion in KPL-3C human breast cancer cells[a]

| | Control | 1 nM E2 | 1 μM TAM | 1 nM ICI |
|---|---|---|---|---|
| Cell no. (×10^6) | 1.1 ± 0.1 | 1.1 ± 0.2 | 0.9 ± 0.2 | 0.9 ± 0.1 |
| PTHrP concentration (pM) | 8.7 ± 0.2 | 7.4 ± 0.7 | 10.5 ± 0.3 | 10.6 ± 0.4 |
| PTHrP secretion[b] | 7.9 ± 0.2 | 6.7 ± 0.6 | 11.7 ± 0.3 | 11.8 ± 0.5 |
| Percentage of control | 100 ± 2 | 83 ± 7 | 144 ± 4 | 146 ± 6 |

[a]Semiconfluent KPL-3C cells were incubated with E2-eliminated medium supplemented with various concentrations of agents for 48 h. After incubation, the conditioned medium was collected and the cell number was counted. The concentration of PTHrP was measured by an IRMA. Values represent means ± s.d. [b]PTHrP secretion (femtomoles) per $10^6$ cells per 48 h was calculated as described in Materials and methods.

control ± s.d.: 82 ± 13 for 1 nM, $P < 0.05$; 67 ± 0 for 10 nM, $P < 0.01$; 68 ± 3 for 100 nM, $P < 0.01$). In contrast, 0.1–10 μM TAM significantly stimulated PTHrP secretion (138 ± 17 for 0.1 μM, 143 ± 3 for 1.0 μM and 118 ± 1 for 10 μM; $P < 0.01$ in each comparison). PTHrP secretion was also significantly stimulated by 1.0–100 nM ICI in a dose-dependent manner (143 ± 0 for 1.0 nM, 157 ± 7 for 10 nM and 205 ± 12 for 100 nM; $P < 0.01$ in each comparison).

Representative experimental data on the number of cells per well, PTHrP concentration measured by an IRMA, PTHrP secretion per $10^6$ cells per 48 h and its percentage of control are shown in Table 1. Because the semiconfluent KPL-3C cells grew slowly, the effects of $E_2$ and antioestrogens on the cell proliferation were limited.

### E2 blocks the stimulation of PTHrP secretion by antioestrogens

Simultaneous addition of 1 nM $E_2$ to the medium completely blocked the stimulative effect of 1.0 μM TAM on PTHrP secretion from KPL-3C cells (Figure 2A). No significant difference in PTHrP secretion was seen between the $E_2$-treated group and the $E_2$ plus TAM-treated group (79 ± 9 and 78 ± 10 respectively). Simultaneous addition of 1 nM $E_2$ also completely blocked the stimulative effect of 1 nM ICI on PTHrP secretion (Figure 2B), with no difference in PTHrP secretion again being seen between the $E_2$-treated group and the $E_2$ plus ICI-treated group (80 ± 4 and 80 ± 2 respectively).

**Figure 2** $E_2$ blocked the stimulative effect of antioestrogens on PTHrP secretion from KPL-3C cells. **(A)** Simultaneous addition of 1 nM $E_2$ to the medium blocked the effect of TAM on PTHrP secretion. **(B)** Simultaneous addition of $E_2$ blocked the effect of ICI. Values represent the mean percentages of control ± s.d. $^{**}P < 0.01$ in comparison with control

### Effects of $E_2$ and antioestrogens on cell cycle

As shown in Table 2, 1–100 nM $E_2$ significantly increased the percentage of cells during the S and $G_2$/M phases ($P < 0.05$ or $P < 0.01$ in each comparison) and decreased the percentage during the $G_0/G_1$ phase ($P < 0.01$ in all comparisons). In contrast, 0.1–10 μM TAM significantly increased the percentage of cells during the $G_0/G_1$ phase and significantly decreased the S-phase fraction ($P < 0.01$ in all comparisons) in a dose-dependent manner. In addition, 1–100 nM ICI significantly increased the percentage of cells during the $G_0/G_1$ phase and decreased the S-phase fraction in a dose-dependent manner ($P < 0.01$ in all comparisons).

### $E_2$ reverses the cytostatic effect of antioestrogens

As shown in Table 3, simultaneous addition of 1 nM $E_2$ to the medium completely reversed the $G_1$–S block by 1 μM TAM. No difference was seen between the control group and the $E_2$ plus TAM-treated group in the percentage of cells during each cell cycle phase. Furthermore, the $G_1$–S block by 1 nM ICI was also reversed by simultaneous addition of 1 nM $E_2$. No difference in the percentage of cells was seen between the $E_2$-treated group and the $E_2$ plus ICI-treated group during each cell cycle phase.

### Reproducibility of the experimental results

In the separate experiments, similar findings described above were reproducibly observed. Among the experiments, the coefficients of variation of percentage of control of PTHrP secretion and of percentage of cells during a $G_0/G_1$ phase after the treatment with each agent were less than 10%. For example, percentages of control of PTHrP secretion treated with 1 nM $E_2$ in three separate experiments were 82 ± 13 as shown in Figure 1, 79 ± 9 in Figure 2A and

© Cancer Research Campaign 1997 

**Table 2** Effects of oestradiol and antioestrogens on the cell cycle progression of KPL-3C cells[a]

| Agent | Percentage of cells during each cell cycle phase | | |
|---|---|---|---|
| | $G_0/G_1$ | S | $G_2/M$ |
| None | 59.6 ± 0.3 | 27.5 ± 0.8 | 12.8 ± 0.5 |
| 1 nM $E_2$ | 55.8 ± 0.3** | 30.2 ± 0.5** | 14.0 ± 0.2** |
| 10 nM $E_2$ | 55.1 ± 0.1** | 31.9 ± 0.5** | 13.1 ± 0.4 |
| 100 nM $E_2$ | 57.0 ± 0.3** | 29.2 ± 0.5** | 13.9 ± 0.2** |
| 0.1 μM TAM | 61.1 ± 0.5** | 24.9 ± 0.9* | 14.1 ± 0.4** |
| 1 μM TAM | 62.7 ± 0.1** | 24.6 ± 0.4** | 12.8 ± 0.3 |
| 10 μM TAM | 67.1 ± 0.3** | 21.2 ± 0.2** | 11.7 ± 0.1* |
| 1 nM ICI | 65.4 ± 0.1** | 21.2 ± 0.2** | 13.4 ± 0.1* |
| 10 nM ICI | 67.7 ± 0.1** | 19.8 ± 0.2** | 12.5 ± 0.2 |
| 100 nM ICI | 69.2 ± 0.2** | 17.6 ± 0.3** | 13.2 ± 0.2 |

[a]Semiconfluent KPL-3C cells were incubated with E2-eliminated medium supplemented with various concentrations of agents for 48 h. After incubation, the cells were trypsinized, harvested, stained with propidium iodine and analysed by flow cytometry. Values represent mean percentages ± s.d. *$P < 0.05$ in comparison with control; **$P < 0.01$ in comparison with control.

**Table 3** Oestradiol reverses the cytostatic effects of antioestrogens in KPL-3C cells[a]

| Agent | Percentage of cells during each cell cycle phase | | |
|---|---|---|---|
| | $G_0/G_1$ | S | $G_2/M$ |
| None | 56.1 ± 0.4 | 31.2 ± 0.6 | 12.8 ± 0.3 |
| 1 nM $E_2$ | 53.6 ± 0.2** | 34.2 ± 0.2** | 12.2 ± 0.4 |
| 1 μM TAM | 61.6 ± 0.1** | 27.4 ± 0.3** | 11.0 ± 0.2** |
| $E_2$+TAM | 57.2 ± 0.2 | 30.2 ± 0.8 | 12.3 ± 0.5 |
| None | 58.4 ± 0.1 | 27.2 ± 0.2 | 14.4 ± 0.2 |
| 1 nM $E_2$ | 57.6 ± 0.2** | 26.8 ± 0.2 | 15.6 ± 0.2** |
| 1 nM ICI | 62.3 ± 0.4** | 23.0 ± 0.4** | 14.7 ± 0.3 |
| $E_2$+ICI | 57.8 ± 0.4 | 27.6 ± 0.5 | 14.6 ± 0.5 |

[a]Semiconfluent KPL-3C cells were incubated with E2-eliminated medium supplemented with the indicated concentrations of each antioestrogen and/or 1 nM E2 for 48 h. After incubation, the collected cells were stained with propidium iodine and analysed by flow cytometry. Values represent mean percentages ± s.d. **$P < 0.01$ in comparison with control.

**Figure 3** Linear correlation of the concentration of PTHrP measured by an IRMA with the concentration of C-terminal PTHrP measured by an RIA in culture medium (the correlation coefficient was 0.98, $P < 0.01$). Conditioned media treated with or without oestrogen or antioestrogens for 48 h were collected, and both PTHrP measurements were performed in each sample as described in Materials and methods

80 ± 4 in Figure 2B. In addition, percentages of cells during $G_0/G_1$ phase treated with 1 nM $E_2$ in three separate experiments were 55.8 ± 0.3 as shown in Table 1 and 53.6 ± 0.2 and 57.6 ± 0.2 in Table 2.

### Correlation between PTHrP concentration measured by an IRMA and by a C-terminal RIA

As shown in Figure 3, PTHrP concentration measured by an IRMA was significantly correlated with that measured by a C-terminal RIA. The concentration of C-terminal PTHrP was slightly higher than the PTHrP concentration measured by an IRMA in each sample. These findings suggest that the amount of C-terminal PTHrP may contain both mature and degraded PTHrP and that the amount of degraded PTHrP may be small in this culture condition.

### DISCUSSION

Although PTHrP was originally isolated from malignancies associated with humoral hypercalcaemia and proved to be the main cause of malignancy-associated hypercalcaemia, recent studies suggest that this protein plays important roles in cell growth and

© Cancer Research Campaign 1997

differentiation in normal organs (Martin et al, 1991). Expression of PTHrP has been demonstrated in most primary breast cancers, and higher expression of PTHrP has been noted in bone metastasis from breast cancer. It has also been suggested that PTHrP may play a role in the development of osteolytic metastasis. In addition, our previous study suggested that PTHrP might play a role in the deposition of microcalcifications in breast cancer tissues (Kurebayashi et al, 1996). Other researchers have suggested certain roles of PTHrP as autocrine and paracrine growth factors in breast cancer cells (Birch et al, 1995). These findings support the hypothesis that PTHrP may be a key factor inducing malignant progression of breast cancer and that suppression of its production and secretion may be effective in the inhibition of breast cancer growth and in the reduction of cancer-related morbidity, such as hypercalcaemia and pathological fractures.

The regulation of PTHrP production and secretion has been widely studied in various cell lines and cell cultures of human normal cells. Very recently, some human breast cancer cell lines have been reported to express PTHrP (Tabuenca et al., 1995; Birch et al, 1996). However, the amount of PTHrP secreted into culture medium from such cell lines is too small to investigate the effects of agents on PTHrP secretion (unpublished data). Recently, we established a novel human breast cancer cell line, KPL-3C, derived from a patient with humoral hypercalcaemia, and preliminary characterization revealed that this cell line possesses both ER and PgR and stably secretes a detectable amount of immunoreactive PTHrP into the culture medium. A glucocorticoid, progestin and vitamin D analogue significantly inhibited PTHrP secretion from KPL-3C cells in a dose-dependent manner (Kurebayashi et al, 1996). This background prompted us to investigate the effects of oestrogen and antioestrogens on PTHrP secretion in this cell line.

Unexpectedly, PTHrP secretion from KPL-3C human breast cancer cells was clearly inhibited by E2 and stimulated by either a non-steroidal antioestrogen, TAM, or a pure steroidal antioestrogen, ICI. To the best of our knowledge, this is the first report demonstrating these interesting phenomena. However, it should be noted that neither PTHrP mRNA level nor proteolytic processing of mature PTHrP was investigated in this study. The concentration of C-terminal PTHrP was linearly correlated with the concentration measured by an IRMA in culture media (Figure 3). This result indicates that both production and secretion of mature PTHrP from KPL-3C cells may be regulated by oestrogen and antioestrogens and that alteration of proteolytic processing of mature PTHrP is unlikely to be a main factor causing this regulation. Further studies are needed to elucidate the regulatory mechanisms of oestrogen and antioestrogens on PTHrP secretion and production in ER-positive breast cancer cells.

Up-regulation of PTHrP secretion by antioestrogens reminds us that antioestrogens sometimes cause a flare phenomenon in patients with advanced breast cancer (Plotkin et al, 1978; Coleman et al, 1988). This unresolved phenomenon might be explained by these data, i.e. a rapid increase in PTHrP secretion from breast cancer cells may activate bone resorption by osteoclasts, which may result in a transient increase in bone pain and may increase the serum calcium level following a rise in the plasma PTHrP level. It is well known that a rapid increase in bone pain and temporary hypercalcaemia are common symptoms of the flare phenomenon. This hypothesis should be clarified both in vitro and in clinical studies.

The addition of 1 nM $E_2$ clearly blocked the stimulation of PTHrP secretion by both antioestrogens. These data suggest that the regulation of PTHrP secretion may be mediated by ER. Interestingly, an inhibitory effect of combined treatment with 1 nM $E_2$ and each antioestrogen on PTHrP secretion was similar to that of treatment with 1 nM $E_2$ alone. The binding affinity of ER for oestrogen and antioestrogens in KPL-3C cells has not yet been investigated. It might be possible that ER in KPL-3C cells is mutated and its binding affinity or signal transduction pathway is different from that of normal ER. Further analysis is needed to clarify this phenomenon. On the other hand, a vitamin D analogue, 22-oxacalcitriol, was reported to act via vitamin D receptor and inhibit the expression and secretion of PTHrP in some cell lines (Inoue et al, 1993; Endo et al, 1994; Dvir et al, 1995). In addition, a progestin, medroxyprogesterone acetate, dose-dependently inhibited PTHrP secretion from the PgR-positive KPL-3C cell line in our previous study (Kurebayashi et al, 1996). Our preliminary study also suggested that oestrogen and antioestrogens did not affect the PTHrP secretion from ER-negative MDA-MB 231 human breast cancer cells (data not shown). These findings suggest that PTHrP secretion may be regulated via each steroid hormone receptor. However, it has been reported that antioestrogens stimulate TGF-β secretion from ER-positive breast cancer cells (Knabbe et al, 1987) and that TGF-β stimulates PTHrP secretion from a primary culture of mammary epithelial cells and canine squamous carcinoma cells (Ferrari et al, 1992; Merryman et al, 1994). It might be possible that antioestrogens stimulate TGF-β secretion from KPL-3C cells and subsequently may increase PTHrP secretion mediated by the TGF-β receptor. The effects of TGF-β and its neutralizing antibody on PTHrP secretion from KPL-3C cells are under investigation. Further studies are clearly needed to clarify and elucidate this phenomenon.

As expected, oestrogen stimulated the cell cycle progression of ER-positive KPL-3C cells and both antioestrogens caused a $G_1$–S block on them. The effects of antioestrogens on the cell cycle progression of ER-positive human breast cancer cells have been extensively studied by many researchers (Sutherland et al, 1986). Antioestrogens are well known to cause a $G_1$–S block and subsequently result in a cytostatic effect on the cells. The experimental results in this study support this phenomenon. Interestingly, although both antioestrogens caused a cytostatic effect on KPL-3C cells, they stimulated PTHrP secretion from the cells. In contrast, a vitamin D analogue, oxacalcitriol, and medroxyprogesterone acetate caused a cytostatic effect and inhibited PTHrP secretion in this cell line (Kurebayashi et al, 1996; unpublished data). These findings suggest that the regulation of PTHrP secretion may not be directly correlated with cell cycle progression. These interesting phenomena should be further studied at the molecular level.

In conclusion, PTHrP secretion from ER-positive KPL-3C cells was inhibited by oestrogen and stimulated by antioestrogens in vitro. These data may explain a flare phenomenon caused by antioestrogens in patients with breast cancer. Studies on the regulatory mechanisms of PTHrP secretion from breast cancer cells may contribute to the development of new strategies not only for the control of malignancy-associated hypercalcaemia but also for the therapy of bone metastasis.

### ACKNOWLEDGEMENTS

The authors would like to thank Dr Robert B Dickson, Lombardi Cancer Center, Georgetown University Medical Center, for his helpful comments on this manuscript as well as faculty members at the Cell Culture Center of Kawasaki Medical School for their

© Cancer Research Campaign 1997

technical assistance. This work was supported in part by a grant from the Ministry of Education, Science, Sports and Culture of Japan and by a Research Project Grant (no. 7-301) from Kawasaki Medical School.

### REFERENCES

Akino K, Ohtsuru A, Yano H, Ozeki S, Namba H, Nakashima M, Ito M, Matsumoto T and Yamashita S (1996) Antisense inhibition of parathyroid hormone-related peptide gene expression reduces malignant pituitary tumor progression and metastases in rat. *Cancer Res* **56**: 77–86

Allinson ET and Drucker DJ (1992) Parathyroid hormone-like peptide shares features with members of the early response gene family: rapid induction by serum, growth factors, and cycloheximide. *Cancer Res* **52**: 3103–3109

Benitez-Verguizas and Esbrit P (1994) Proliferative effect of parathyroid hormone-related protein on the hypercalcemic Walker 256 carcinoma cell line. *Biochem Biophys Res Commun* **198**: 1281–1289

Birch MA, Carron JA, Scott M, Fraser WD and Gallagher JA (1995) Parathyroid hormone (PTH)/PTH-related protein (PTHrP) receptor expression and mitogenic responses in human breast cancer cell lines. *Br J Cancer* **72**: 90–95

Bundred NJ, Ratcliffe WA, Walker RA, Coley S, Morrison JM and Ratcliffe JG (1991) Parathyroid hormone-related protein and hypercalcemia in breast cancer. *Br Med J* **303**: 1506–1509

Burtis WJ, Brady TG, Orloff JJ, Ersbak JB, Warrell RP, Olson BR, Wu TL, Mitnick ME, Broadus AE and Stewart AF (1990) Immunochemical characterization of circulating parathyroid hormone-related protein in patients with humoral hypercalcemia of cancer. *N Engl Med* **322**: 1106–1112

Coleman RE, Whitaker KB, Moss DW, Mashiter G, Fogelman I and Rubens RD (1988) Biochemical prediction of response of bone metastases to treatment. *Br J Cancer* **58**: 205–210

Deftos LJ, Houge-Angeletti R, Chalberg C and Tu S (1989) PTHrP secretion is stimulated by CT and inhibited by CgA peptides. *Endocrinology* **125**: 563–565

Dvir R, Golander A, Jaccard N, Yedwab G, Otremski I, Spirer Z and Weisman (1995) Amniotic fluid and plasma levels of parathyroid hormone-related protein and hormonal modulation of its secretion by amniotic fluid cells. *Eur J Endocrinol* **133**: 277–282

Emly JF, Hughes S, Green E and Ratcliffe WA (1994) Expression and secretion of parathyroid hormone-related protein by a human cancer cell line. *Biochim Biophys Acta* **1220**: 193–198

Endo K, Ichikawa F, Uchiyama Y, Katsumata K, Ohkawa H, Kumaki K, Ogata E and Ikeda K (1994) Evidence for the uptake of a vitamin D analogue (OCT) by a human carcinoma and its effect of suppressing the transcription of parathyroid hormone-related peptide gene *in vivo*. *J Biol Chem* **269**: 32693–32699

Ferrari SL, Rizzoli R and Bonjour JP (1992) Parathyroid hormone-related protein production by primary cultures of mammary epithelial cells. *J Cell Physiol* **150**: 304–311

Grill V, Ho P, Moseley JM, Johnason N, Lee S, Body JJ, Kukreja S and Martin TJ (1991) Parathyroid hormone-related protein: elevated levels both in humoral hypercalcemia of malignancy and in hypercalcemia complicating metastatic breast cancer. *J Clin Endocrinol Metab* **73**: 1309–1315

Henderson J, Sebag M, Rhim J, Goltzman D and Kremer R (1991) Dysregulation of parathyroid hormone-like peptide expression and secretion in a keratinocyte model of tumor progression. *Cancer Res* **51**: 6521–6528

Holt EH, Lu C, Dreyer BE, Dannies PS and Broadus AE (1994) Regulation of parathyroid hormone-related peptide gene expression by estrogen in GH4C1 rat pituitary cells has the pattern of a primary response gene. *J Neurochem* **62**: 1239–1246

Ikeda K, Ohno H, Hane M, Yokoi H, Okada M, Honma T, Yamada A, Tatsumi Y, Tanaka T, Saitoh T, Hirose S, Mori S, Takeuchi Y, Fukumoto S, Terukina S, Iguchi H, Kiriyama T, Ogata E and Matsumoto T (1994) Development of a sensitive two-site immunoradiometric assay for parathyroid hormone-related peptide: evidence for elevated levels in plasma from patients with adult T-cell leukemia/lymphoma and B-cell lymphoma. *J Clin Endocrinol Metab* **79**: 1322–1327

Inoue D, Matsumoto T, Ogata E and Ikeda K (1993) 22-oxacalcitriol, noncalcemic analogue of calcitriol, suppresses both cell proliferation and parathyroid hormone-related peptide gene expression in human T cell lymphotrophic virus, type I-infected T cells. *J Biol Chem* **268**: 16730–16736

Iwamura M, Wu G, Abrahamsson PA, Cockett ATK, Foss KA and Deftos LJ (1994) Parathyroid hormone-related protein: a potential autocrine growth regulator in human prostate cancer cell lines. *Urology* **43**: 675–679

Kaiser SM, Lanevrille P, Bernier SM, Rhim JS, Kremer R and Goltzman D (1992) Enhanced growth of a human keratinocyte cell line induced by antisense RNA for parathyroid hormone-related protein. *J Biol Chem* **267**: 13623–13628

Kasahara H, Tsuchiya M, Adachi R, Hirokawa S, Tanaka S and Tachibana S (1992) Development of a C-terminal-region-specific radioimmunoassay of parathyroid hormone-related protein. *Biomed Res* **13**: 155–161

Knabbe C, Lippman ME, Wakefield LM, Flanders KC, Kasid A, Derynck R and Dickson RB (1987) Evidence that transforming growth factor-β is a hormonally regulated negative growth factor in human breast cancer cells. *Cell* **48**: 417–428

Kremer R, Karaplis AC, Henderson J, Gulliver W, Banville D, Hendy GN and Goltzman D (1991) Regulation of parathyroid hormone-like peptide in cultured normal human keratinocytes. *J Clin Invest* **87**: 884–893

Kurebayashi J, Kurosumi M and Sonoo H (1996) A new human breast cancer cell line, KPL-3C secretes parathyroid hormone-related protein and produces tumours associated with microcalcifications in nude mice. *Br J Cancer* **74**: 200–207

Li X and Drucker DJ (1994) Parathyroid hormone-related protein is a downstream target for *ras* and *src* activation. *J Biol Chem* **269**: 6263–6266

Luparello C, Burtis WJ, Raue F, Birch MA and Gallagher JA (1995) Parathyroid hormone-related peptide and 8701-BC breast cell growth and invasion *in vitro*: evidence for growth-inhibiting and invasion-promoting effects. *Mol Cell Endocrinol* **111**: 225–232

Martin TJ, Moseley JM and Gillespie MT (1991) Parathyroid hormone-related protein: biochemistry and molecular biology. *Crit Rev Biochem Mol Biol* **26**: 377–395

Merryman JI, DeWille JW, Werkmeister JR, Capen CC and Rosol TJ (1994) Effects of transforming growth factor-β on parathyroid hormone-related protein production and ribonucleic acid expression by a squamous carcinoma cell line *in vitro*. *Endocrinology* **134**: 2424–2430

Moseley JM, Kubota M, Diefenbach-Jagger H, Wettenhall REH, Kemp BE, Suva LJ, Rodda CP, Ebeling PR, Hudson PJ, Zajac JD and Martin TJ (1987) Parathyroid hormone-related protein purified from a human lung cancer cell line. *Proc Natl Acad Sci USA* **84**: 5048–5052

Paspaliaris V, Vargas SJ, Gillespie MT, Williams ED, Danks JA, Moseley JM, Story ME, Pennefather JN, Leaver DD and Martin TJ (1992) Oestrogen enhancement of the myometrial response to exogenous parathyroid hormone-related protein (PTHrP) and tissue localization of endogenous PTHrP and its mRNA in the virgin rat uterus. *J Endocrinol* **134**: 415–425

Plotkin D, Lechner JJ, Jung WE and Rosen PJ (1978) Tamoxifen flare in advanced breast cancer. *JAMA* **240**: 2644–2646

Powell GJ, Southby J, Danks JA, Stillwell RG, Hayman JA, Henderson MA, Bennett RC and Martin TJ (1991) Localization of parathyroid hormone-related protein in breast cancer metastases: increased incidence in bone compared with other sites. *Cancer Res* **51**: 3059–3061

Ratcliffe WA, Hutchesson ACJ, Bundred NJ and Ratcliffe JG (1992) Role of assays for parathyroid-hormone-related protein in investigation of hypercalcemia. *Lancet* **339**: 164–167

Rizzoli R, Aubert ML, Sappino AP and Bonjour JP (1994) Cyclic AMP increases the release of parathyroid hormone-related protein from a lung-cancer cell line. *Int J Cancer* **56**: 422–426

Scholl SM, Huff KK and Lippman ME (1983) Antiestrogenic effects of LY117018 in MCF-7 cells. *Endocrinology* **113**: 611–617

Sebag M, Henderson J, Goltzman D and Kremer R (1994) Regulation of parathyroid hormone-related peptide production in normal human mammary epithelial cells *in vitro*. *Am J Physiol* **267**: C723–C730

Southby J, Kissin MW, Danks JA, Hayman JA, Moseley JM, Henderson MA, Bennett RC and Martin TJ (1990) Immunohistochemical localization of parathyroid hormone-related protein in human breast cancer. *Cancer Res* **50**: 7710–7716

Sutherland RL, Reddel RR, Murphy LC and Taylor IW (1986) Effects of antiestrogens on cell cycle progression. In *Estrogen/Antiestrogen Action and Breast Cancer Therapy*, Jordan VC. (ed.), pp. 265–279. The University of Wisconsin Press: Wisconsin

Suva LJ, Winslow GA, Wettenhall REH, Hammonds RG, Moseley JM, Diefenbach-Jagger H, Rodda CP, Kemp BE, Rodriguez H, Chen EY, Hudson PJ, Martin TJ and Wood WI (1987) A parathyroid hormone-related protein implicated in malignant hypercalcemia: cloning and expression. *Science* **237**: 893–896

© Cancer Research Campaign 1997

Tabuenca A, Mohan S, Garberoglio CA, Borgen PI, Rosol T and Linkhart TA (1995) Parathyroid hormone-related protein: primary osteolytic factor produced by breast tumor cells *in vitro*? *World J Surg* **19**: 292–297

Thiede MA (1989) The mRNA encoding a parathyroid hormone-related peptide is produced in mammary tissue in response to deviations in serum prolactin. *Mol Endocrinol* **3**: 1443–1447

Thiede MA, Harm SC, Hasson DM and Gardner RM (1991) *In vivo* regulation of parathyroid hormone-related peptide messenger ribonucleic acid in the rat uterus by 17β-estradiol. *Endocrinology* **128**: 2317–2323

Vargas SJ, Gillespie MT, Powell GJ, Southby J, Danks JA, Moseley JM and Martin TJ (1992) Localization of parathyroid hormone-related protein mRNA expression in breast cancer and metastatic lesions by *in situ* hybridization. *J Bone Miner Res* **7**: 971–979

© Cancer Research Campaign 1997


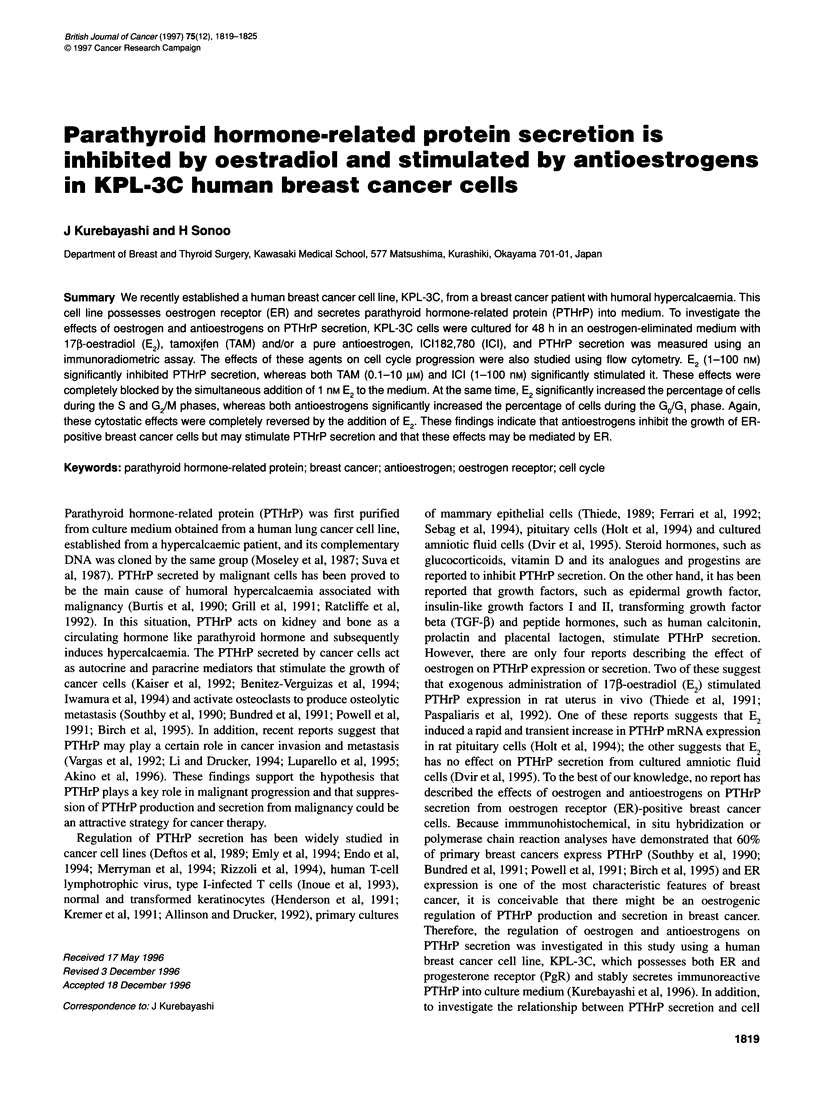

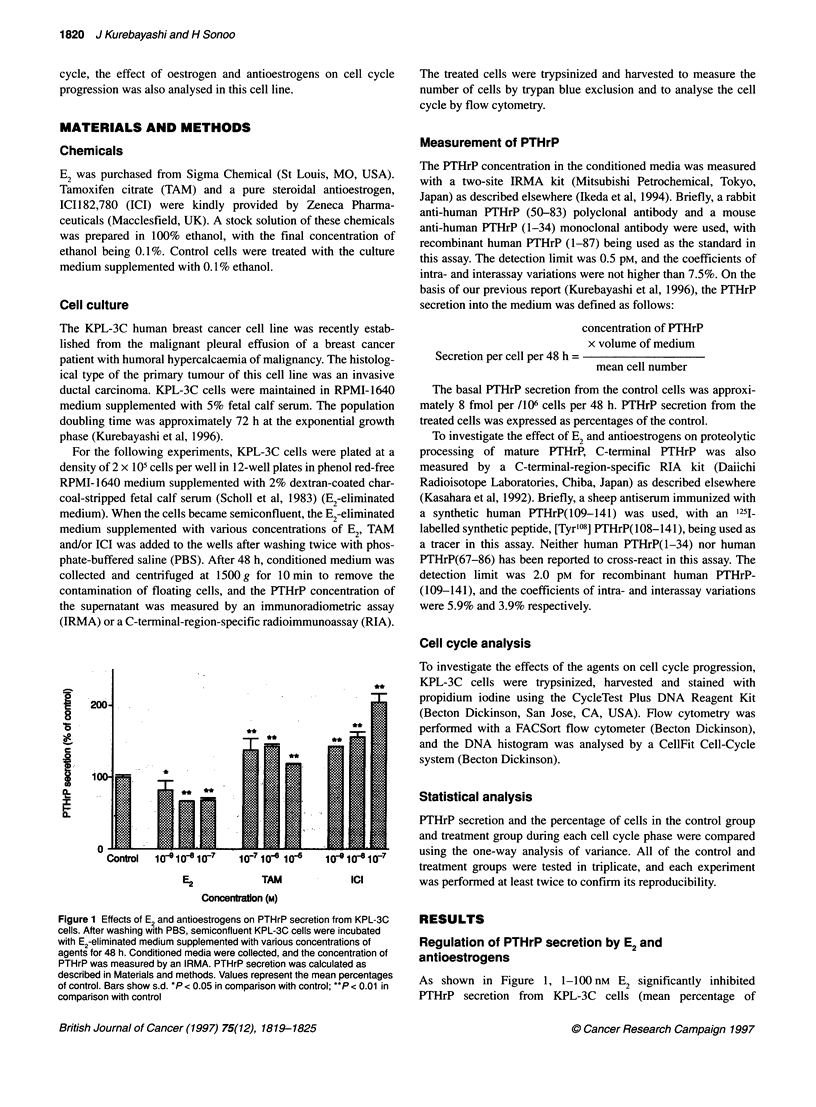

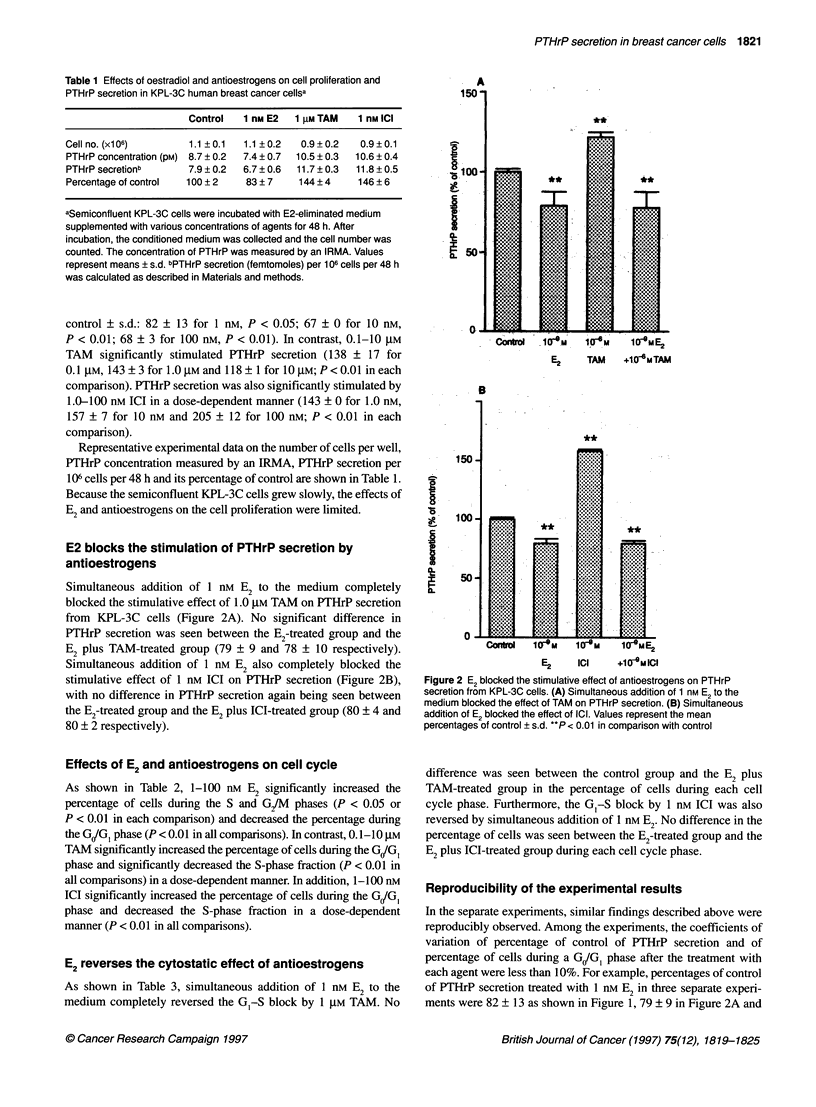

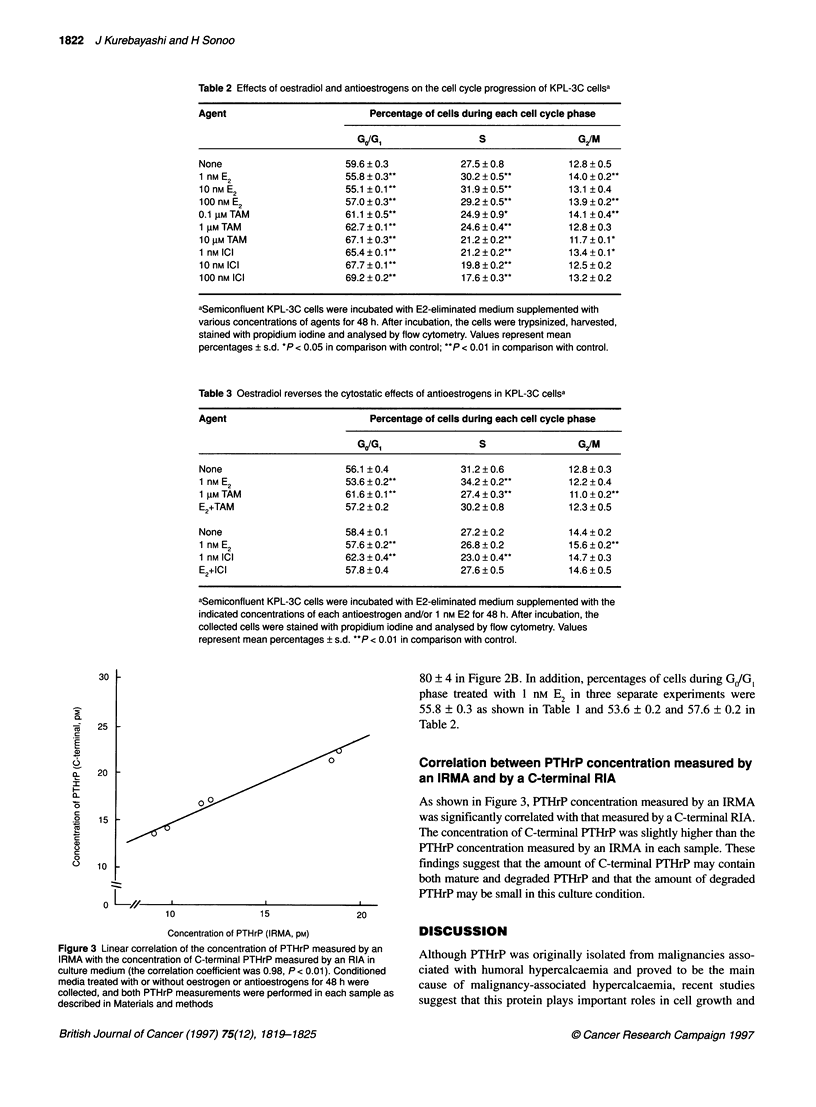

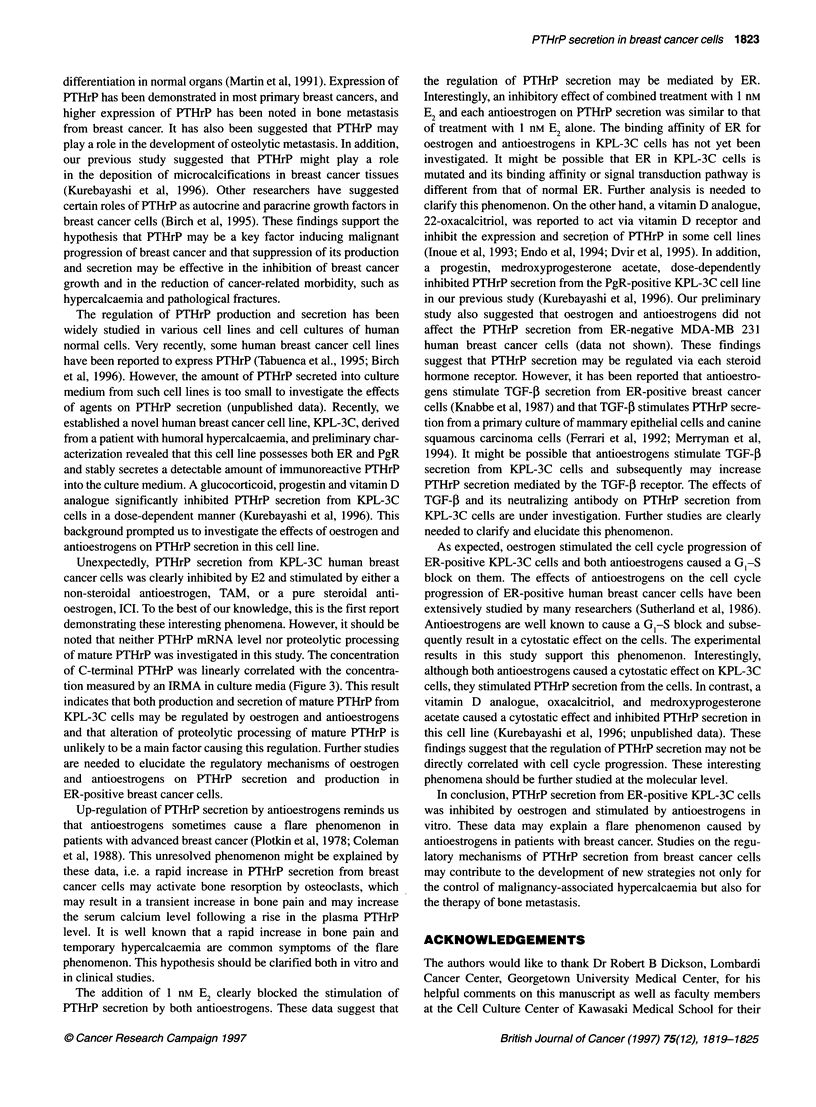

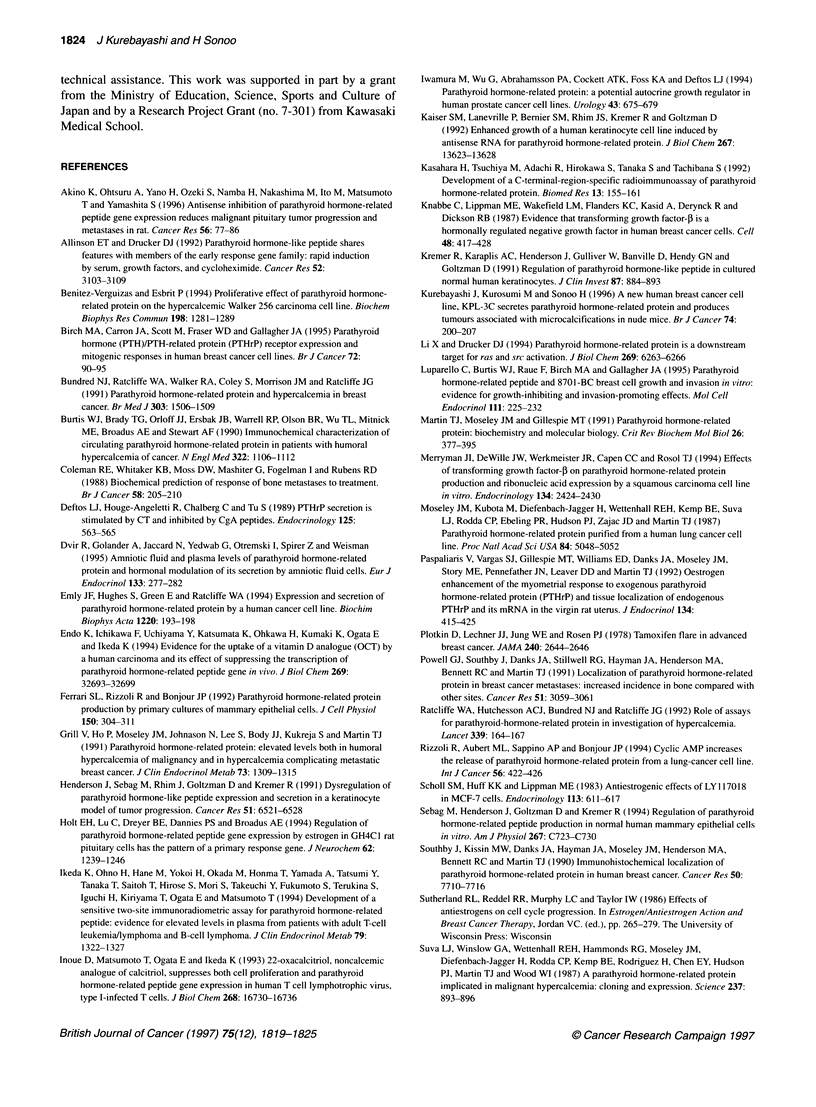

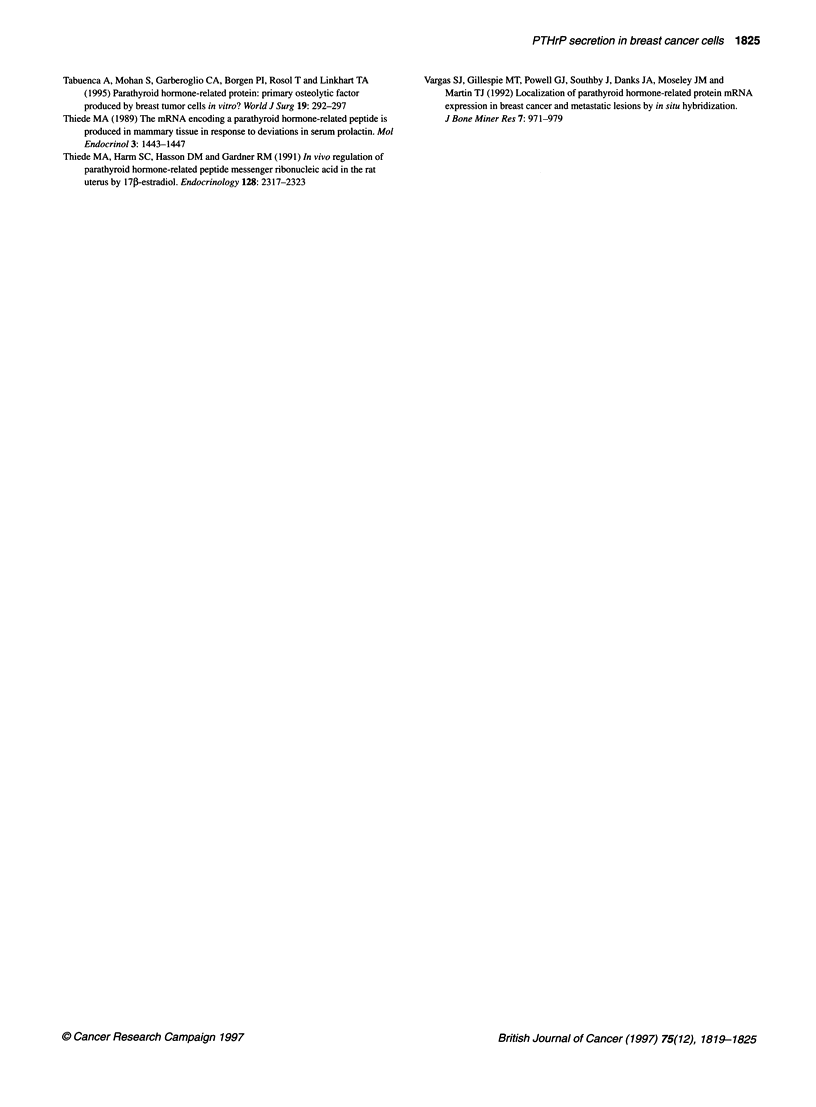


## References

[OCR_00635] Akino K., Ohtsuru A., Yano H., Ozeki S., Namba H., Nakashima M., Ito M., Matsumoto T., Yamashita S. (1996). Antisense inhibition of parathyroid hormone-related peptide gene expression reduces malignant pituitary tumor progression and metastases in the rat.. Cancer Res.

[OCR_00641] Allinson E. T., Drucker D. J. (1992). Parathyroid hormone-like peptide shares features with members of the early response gene family: rapid induction by serum, growth factors, and cycloheximide.. Cancer Res.

[OCR_00647] Benítez-Verguizas J., Esbrit P. (1994). Proliferative effect of parathyroid hormone-related protein on the hypercalcemic Walker 256 carcinoma cell line.. Biochem Biophys Res Commun.

[OCR_00652] Birch M. A., Carron J. A., Scott M., Fraser W. D., Gallagher J. A. (1995). Parathyroid hormone (PTH)/PTH-related protein (PTHrP) receptor expression and mitogenic responses in human breast cancer cell lines.. Br J Cancer.

[OCR_00658] Bundred N. J., Ratcliffe W. A., Walker R. A., Coley S., Morrison J. M., Ratcliffe J. G. (1991). Parathyroid hormone related protein and hypercalcaemia in breast cancer.. BMJ.

[OCR_00663] Burtis W. J., Brady T. G., Orloff J. J., Ersbak J. B., Warrell R. P., Olson B. R., Wu T. L., Mitnick M. E., Broadus A. E., Stewart A. F. (1990). Immunochemical characterization of circulating parathyroid hormone-related protein in patients with humoral hypercalcemia of cancer.. N Engl J Med.

[OCR_00669] Coleman R. E., Whitaker K. B., Moss D. W., Mashiter G., Fogelman I., Rubens R. D. (1988). Biochemical prediction of response of bone metastases to treatment.. Br J Cancer.

[OCR_00674] Deftos L. J., Hogue-Angeletti R., Chalberg C., Tu S. (1989). PTHrP secretion is stimulated by CT and inhibited by CgA peptides.. Endocrinology.

[OCR_00679] Dvir R., Golander A., Jaccard N., Yedwab G., Otremski I., Spirer Z., Weisman Y. (1995). Amniotic fluid and plasma levels of parathyroid hormone-related protein and hormonal modulation of its secretion by amniotic fluid cells.. Eur J Endocrinol.

[OCR_00686] Emly J. F., Hughes S., Green E., Ratcliffe W. A. (1994). Expression and secretion of parathyroid hormone-related protein by a human cancer cell line.. Biochim Biophys Acta.

[OCR_00691] Endo K., Ichikawa F., Uchiyama Y., Katsumata K., Ohkawa H., Kumaki K., Ogata E., Ikeda K. (1994). Evidence for the uptake of a vitamin D analogue (OCT) by a human carcinoma and its effect of suppressing the transcription of parathyroid hormone-related peptide gene in vivo.. J Biol Chem.

[OCR_00698] Ferrari S. L., Rizzoli R., Bonjour J. P. (1992). Parathyroid hormone-related protein production by primary cultures of mammary epithelial cells.. J Cell Physiol.

[OCR_00703] Grill V., Ho P., Body J. J., Johanson N., Lee S. C., Kukreja S. C., Moseley J. M., Martin T. J. (1991). Parathyroid hormone-related protein: elevated levels in both humoral hypercalcemia of malignancy and hypercalcemia complicating metastatic breast cancer.. J Clin Endocrinol Metab.

[OCR_00709] Henderson J., Sebag M., Rhim J., Goltzman D., Kremer R. (1991). Dysregulation of parathyroid hormone-like peptide expression and secretion in a keratinocyte model of tumor progression.. Cancer Res.

[OCR_00714] Holt E. H., Lu C., Dreyer B. E., Dannies P. S., Broadus A. E. (1994). Regulation of parathyroid hormone-related peptide gene expression by estrogen in GH4C1 rat pituitary cells has the pattern of a primary response gene.. J Neurochem.

[OCR_00720] Ikeda K., Ohno H., Hane M., Yokoi H., Okada M., Honma T., Yamada A., Tatsumi Y., Tanaka T., Saitoh T. (1994). Development of a sensitive two-site immunoradiometric assay for parathyroid hormone-related peptide: evidence for elevated levels in plasma from patients with adult T-cell leukemia/lymphoma and B-cell lymphoma.. J Clin Endocrinol Metab.

[OCR_00730] Inoue D., Matsumoto T., Ogata E., Ikeda K. (1993). 22-Oxacalcitriol, a noncalcemic analogue of calcitriol, suppresses both cell proliferation and parathyroid hormone-related peptide gene expression in human T cell lymphotrophic virus, type I-infected T cells.. J Biol Chem.

[OCR_00737] Iwamura M., Abrahamsson P. A., Foss K. A., Wu G., Cockett A. T., Deftos L. J. (1994). Parathyroid hormone-related protein: a potential autocrine growth regulator in human prostate cancer cell lines.. Urology.

[OCR_00742] Kaiser S. M., Laneuville P., Bernier S. M., Rhim J. S., Kremer R., Goltzman D. (1992). Enhanced growth of a human keratinocyte cell line induced by antisense RNA for parathyroid hormone-related peptide.. J Biol Chem.

[OCR_00754] Knabbe C., Lippman M. E., Wakefield L. M., Flanders K. C., Kasid A., Derynck R., Dickson R. B. (1987). Evidence that transforming growth factor-beta is a hormonally regulated negative growth factor in human breast cancer cells.. Cell.

[OCR_00761] Kremer R., Karaplis A. C., Henderson J., Gulliver W., Banville D., Hendy G. N., Goltzman D. (1991). Regulation of parathyroid hormone-like peptide in cultured normal human keratinocytes. Effect of growth factors and 1,25 dihydroxyvitamin D3 on gene expression and secretion.. J Clin Invest.

[OCR_00766] Kurebayashi J., Kurosumi M., Sonoo H. (1996). A new human breast cancer cell line, KPL-3C, secretes parathyroid hormone-related protein and produces tumours associated with microcalcifications in nude mice.. Br J Cancer.

[OCR_00773] Li X., Drucker D. J. (1994). Parathyroid hormone-related peptide is a downstream target for ras and src activation.. J Biol Chem.

[OCR_00777] Luparello C., Burtis W. J., Raue F., Birch M. A., Gallagher J. A. (1995). Parathyroid hormone-related peptide and 8701-BC breast cancer cell growth and invasion in vitro: evidence for growth-inhibiting and invasion-promoting effects.. Mol Cell Endocrinol.

[OCR_00783] Martin T. J., Moseley J. M., Gillespie M. T. (1991). Parathyroid hormone-related protein: biochemistry and molecular biology.. Crit Rev Biochem Mol Biol.

[OCR_00788] Merryman J. I., DeWille J. W., Werkmeister J. R., Capen C. C., Rosol T. J. (1994). Effects of transforming growth factor-beta on parathyroid hormone-related protein production and ribonucleic acid expression by a squamous carcinoma cell line in vitro.. Endocrinology.

[OCR_00795] Moseley J. M., Kubota M., Diefenbach-Jagger H., Wettenhall R. E., Kemp B. E., Suva L. J., Rodda C. P., Ebeling P. R., Hudson P. J., Zajac J. D. (1987). Parathyroid hormone-related protein purified from a human lung cancer cell line.. Proc Natl Acad Sci U S A.

[OCR_00802] Paspaliaris V., Vargas S. J., Gillespie M. T., Williams E. D., Danks J. A., Moseley J. M., Story M. E., Pennefather J. N., Leaver D. D., Martin T. J. (1992). Oestrogen enhancement of the myometrial response to exogenous parathyroid hormone-related protein (PTHrP), and tissue localization of endogenous PTHrP and its mRNA in the virgin rat uterus.. J Endocrinol.

[OCR_00811] Plotkin D., Lechner J. J., Jung W. E., Rosen P. J. (1978). Tamoxifen flare in advanced breast cancer.. JAMA.

[OCR_00815] Powell G. J., Southby J., Danks J. A., Stillwell R. G., Hayman J. A., Henderson M. A., Bennett R. C., Martin T. J. (1991). Localization of parathyroid hormone-related protein in breast cancer metastases: increased incidence in bone compared with other sites.. Cancer Res.

[OCR_00821] Ratcliffe W. A., Hutchesson A. C., Bundred N. J., Ratcliffe J. G. (1992). Role of assays for parathyroid-hormone-related protein in investigation of hypercalcaemia.. Lancet.

[OCR_00826] Rizzoli R., Aubert M. L., Sappino A. P., Bonjour J. P. (1994). Cyclic AMP increases the release of parathyroid hormone-related protein from a lung-cancer cell line.. Int J Cancer.

[OCR_00831] Scholl S. M., Huff K. K., Lippman M. E. (1983). Antiestrogenic effects of LY 117018 in MCF-7 cells.. Endocrinology.

[OCR_00835] Sebag M., Henderson J., Goltzman D., Kremer R. (1994). Regulation of parathyroid hormone-related peptide production in normal human mammary epithelial cells in vitro.. Am J Physiol.

[OCR_00840] Southby J., Kissin M. W., Danks J. A., Hayman J. A., Moseley J. M., Henderson M. A., Bennett R. C., Martin T. J. (1990). Immunohistochemical localization of parathyroid hormone-related protein in human breast cancer.. Cancer Res.

[OCR_00853] Suva L. J., Winslow G. A., Wettenhall R. E., Hammonds R. G., Moseley J. M., Diefenbach-Jagger H., Rodda C. P., Kemp B. E., Rodriguez H., Chen E. Y. (1987). A parathyroid hormone-related protein implicated in malignant hypercalcemia: cloning and expression.. Science.

[OCR_00865] Tabuenca A., Mohan S., Garberoglio C. A., Borgen P. I., Rosol T., Linkhart T. A. (1995). Parathyroid hormone-related protein: primary osteolytic factor produced by breast tumor cells in vitro?. World J Surg.

[OCR_00875] Thiede M. A., Harm S. C., Hasson D. M., Gardner R. M. (1991). In vivo regulation of parathyroid hormone-related peptide messenger ribonucleic acid in the rat uterus by 17 beta-estradiol.. Endocrinology.

[OCR_00870] Thiede M. A. (1989). The mRNA encoding a parathyroid hormone-like peptide is produced in mammary tissue in response to elevations in serum prolactin.. Mol Endocrinol.

[OCR_00880] Vargas S. J., Gillespie M. T., Powell G. J., Southby J., Danks J. A., Moseley J. M., Martin T. J. (1992). Localization of parathyroid hormone-related protein mRNA expression in breast cancer and metastatic lesions by in situ hybridization.. J Bone Miner Res.

